# Traditional bone setting in Nigeria from the perspectives of patients and physiotherapists— clinical insights for low back pain management

**DOI:** 10.1186/s12906-025-04966-z

**Published:** 2025-07-03

**Authors:** Mishael Adje, Sven Karstens, Chidozie Mbada, Jost Steinhäuser

**Affiliations:** 1 Department of Health, LUNEX University of Applied Sciences, Differdange, Luxembourg; 2 Luxembourg Health and Sport Sciences Research Institute, Differdange, Luxembourg; 3 Therapeutic Sciences, Department of Computer Science, Trier University of Applied Sciences, Trier, Germany; 4 Department of Health Professions, Manchester Metropolitan University, Manchester, UK; 5 Institute of Family Medicine, University of Luebeck, Luebeck, Germany

**Keywords:** Physiotherapy, Low back pain, Traditional Bone Setters, Integrative Care, Professional physiotherapy, Musculoskeletal physiotherapy

## Abstract

**Background:**

Traditional bone setting (TBS) remains a prevalent healthcare practice in Nigeria, offering complementary treatments for musculoskeletal conditions such as low back pain (LBP). This study explores the perspectives of both patients and physiotherapists regarding TBS and its implications for the management of LBP.

**Methods:**

A qualitative research approach was employed, utilizing semi-structured interviews with 25 participants (13 patients who had utilized TBS services for LBP, and 12 physiotherapists). Theoretical sampling was employed in participant recruitment until saturation. Recordings were transcribed and thematic analysis was conducted as a secondary analysis. Reporting was informed by the Consolidated Criteria for Reporting Qualitative Research (COREQ).

**Results:**

Eleven (11) participants were female and 14 were male; with a mean age of 35 years. Five themes were identified from this study: driving impetus for TBS, influencing perceptions with information, turning to TBS as a final recourse, exploring the primary alternative, and integrating TBS. The shift towards TBS for LBP stems from deficiencies in prevailing healthcare practices in Nigeria. Patients and physiotherapists hold varying perspectives regarding cultural significance, and effectiveness and safety of TBS compared to professional physiotherapy interventions. Accessibility, affordability, and perceived efficacy are common facilitators for the patronage of TBS among patients, while others were hesitant and viewed it as a last resort. Negative perception of physiotherapists about TBS for LBP were based on concerns regarding the lack of scientific evidence, standardized practices, and potential complications associated with the procedures.

**Conclusion:**

Participants consider TBS viable when professional physiotherapy fails, citing expectations for therapist-guided techniques, affordability, and cultural factors. Physiotherapists remain cautious, stressing the need for evidence-based care and noting severe TBS complications. Some participants advocate for integrating TBS with professional healthcare through collaboration and better communication. This study, supported by literature, highlights the potential for TBS integration, with open communication and training fostering collaboration. Future studies could investigate the practicality of this integration, prioritizing culturally appropriate, safe, and effective approaches to LBP management.

**Supplementary Information:**

The online version contains supplementary material available at 10.1186/s12906-025-04966-z.

## Introduction

Low back pain (LBP) is among the most frequent reasons for visits to outpatient physiotherapy clinics worldwide and also in Nigeria [1–3]. LBP is also highly prevalent, affecting about 60% to 90% of the population at one point in their lives or another [4]. Studies show that LBP is a leading cause of disability and has a significant impact on productivity and personal life [5–7]. Despite treatment efforts, 35% of acute LBP cases turn chronic and the prevalence and recurrence levels remain high, even exponentially increasing which translates significantly in terms of the large population of nearly 250 million Nigerians [8–10]. In Nigeria, patients have the option to access two available systems of health care, traditional and professional physiotherapy practice. Although these practices are often viewed as rivals rather than complementary, the choice ultimately depends on the patient's personal preference [11, 12].

Physiotherapy for LBP in Nigeria is largely professional, involving use of a variety of modalities for light, thermal, mechanical, and electromagnetic energies, manual techniques, exercises, and patient education, among others. Oftentimes, these interventions are used in combination or separately to treat patients with LBP [13, 14]. On the other hand, traditional remedies for LBP, especially by traditional bone setting (TBS), are still common in many countries around the world such as Nigeria, Brazil, China, Korea, Europe and Japan [15–18]. Variations exist in the method and scope of TBS for musculoskeletal conditions but with similarities in the use of herbs.

TBS practices have long been embedded in the healthcare landscape of Nigeria, offering complementary approaches to managing musculoskeletal ailments, including LBP and enjoying high patronage especially in the rural settings in Nigeria [19–21]. TBS is sometimes referred to as a form of Traditional Medicine and often passed ‘from father to child’ or by a practitioner usually experienced in such trade. TBS encompasses some form of lay joint manipulation or healing using herbs and massage typically unlicensed by state and lack provincial licensure by a health ministry or department of health. [21, 22].

TBS has been associated with some complications such as mal-union, non-union, gangrene, chronic osteomyelitis, Volkmann’s ischemic contracture and joint stiffness [23–25]. A study by Odatuwa-Omagbemi et al. [23] concluded that major complications like non-union of fractures, mal-unions, chronic neglected joint dislocations and upper limb gangrene in two children were associated with TBS. Despite this, wide patronage and a positive attitude towards TBS for MSK disorders cutting across various sociodemographic, levels of education and exposure remains [23, 24]. Literature reveals that TBS practitioners are highly trusted and widely patronized by the Nigerian society. In certain areas in southern Nigeria, these practitioners provide about 70%–90% of initial musculoskeletal care [23, 26, 27]. Integrating TBS into the mainstream medical practice has been a topic of debate, especially among orthopedic practitioners and physiotherapists whose domain TBS arguably encroaches [26]. Opinions on integrating TBS into mainstream healthcare stem from the ease of accessing these practitioners in rural regions, lower cost of care, social factors and cultural beliefs [26, 28–30].

Advancements in medicine and physiotherapy that are witnessed in many advanced countries, are yet to be attained in low-and-middle-income countries (LMICs) [29, 31]. As a result, TBS continues to play a significant role in addressing healthcare needs, particularly in rural and medically underserved communities with limited access to professional healthcare services [12]. Since early 1970, the World Health Organization (WHO) has supported using traditional healing approaches to address the limited accessibility to healthcare in LMICS, recommending its integration into the professional healthcare system, while regarding its practitioners as the backbone of primary healthcare system in medically underserved settings [26, 27, 32]. However, the coexistence of traditional and professional healthcare systems raises important questions in the minds of clinicians regarding the efficacy, safety, and cultural significance of TBS in the context of LBP management [26]. Patients on the other hand pay repeated visits to these care centers and generally find it a useful option despite having in some cases mixed outcomes [20]. Hence, the need for a holistic perspective of the existing situation.

### Rationale

Previous studies exploring the perception of physiotherapists and patients to the use of TBS in the management of LBP have been limited to specific regions in Nigeria and have adopted quantitative methods [20, 24]. This study holds significant implications for healthcare policy, practice, and education in Nigeria, where the integration of traditional and professional healthcare systems presents unique opportunities and challenges. By shedding light on the perspectives of both patients and healthcare professionals, this analysis seeks to contribute to a nuanced understanding of TBS and its role in LBP management, ultimately guiding the development of culturally sensitive and evidence-based approaches to healthcare delivery in Nigeria and similar contexts.

### Objective

This manuscript explores the perspectives of both patients and physiotherapists on TBS and its implications for the management of LBP in Nigeria. By delving into the experiences, beliefs, and practices surrounding TBS, as well as contrasting them with the perspectives of trained physiotherapy professionals, this study aims to provide valuable clinical insights for optimizing LBP management strategies in Nigeria and beyond.

## Method

This study utilised a qualitative research approach based on the grounded theory methodology. This study seeks to elucidate the diverse viewpoints and perceptions surrounding TBS uncovering common themes, challenges, and opportunities associated with TBS as a healthcare modality for LBP. Semi-structured telephone interviews were conducted by MA (male) with patients, some who have sought TBS services for LBP and physiotherapists practicing in Nigeria [33]. The study employed thematic analysis and reporting was informed by the Consolidated Criteria for Reporting Qualitative Research (COREQ) [34]. This study is a secondary analysis drawn from a primary study investigating attitudes and perceptions towards a stratified model of care for low back pain. The method is detailed and shown in a published manuscript by Adje et al. [35].

The research team comprised researchers with extensive experience in qualitative research, healthcare services, and musculoskeletal health. Participant recruitment involved theoretical sampling [36], with physiotherapists and patients in Nigeria being contacted based on set inclusion criteria. Physiotherapists need to be licensed and registered by the Medical Rehabilitation Therapist Board of Nigeria, with entry-level qualifications and above. Patients were required to have a diagnosis of non-specific LBP, fluency in English and be above 18 years old. Patients with severe underlying conditions such as cancer were excluded. Recruitment continued until empiric data saturation was achieved [37].

### Data collection

The data collection process involved conducting interviews over the phone. The interview guides tailored for patients and physiotherapists were prepared in four phases: brainstorming, collection, sorting, and examining questions. For the patient guideline, the questions were simplified for clarity, then categorized for thematic structure. A patient with LBP reviewed them and included patient perspective [38]. Four key questions were developed, with additional maintenance and follow-up questions. Patient guideline available here: (https://bmjopen.bmj.com/content/bmjopen/12/6/e059736/ DC1/embed/inline-supplementary-material-1.pdf?download = true). For the physiotherapist guideline, questions were revised to reflect physiotherapists' perspectives and reviewed by two experienced physiotherapists leading to modifications following the approach of Pepper et al. [39]. Four key questions, along with maintenance and follow-up questions, were developed. Physiotherapist guideline available here: (https://bmjopen.bmj.com/content/bmjopen/12/6/e059736/DC2/embed/inline-supplementary-material-2.pdf?download=true). Details on this has been described elsewhere in Adje et al., [35]. Individual verbal consent was received, and interviews were recorded with handwritten notes taken. Notes were summarized back to the participants after each interview and memos were written about each interview. This process was repeated for the various rounds until saturation, where no new categories emerged [37]. The transcription and pseudonymization details along with analysis are described elsewhere [35].

### Data analysis

This secondary analysis was used to provide background context and a better understanding of the phenomenon of TBS in Nigeria. It was also chosen to consider experiences including those relating to practice [40]. Transcripts were read and re-read by two members of the research team. Categories were identified inductively leading to a coding agenda (Appendix 1) further detailed elsewhere [35]. Areas of discrepancies were resolved. Code labels were lifted directly from participants' quotes identifying descriptive categories first [41]. Higher-level abstract categories were systematically identified as coding progressed. They were then tested against the data and consolidated repeatedly. The RQDA package in the R software was used for the coding process [42].

### Ethical approval

Ethical approval was obtained from the Trier University of Applied Sciences and the Obafemi Awolowo University Teaching Hospital, Ile-Ife Nigeria (registration ID: IRB/IEC/0004553). All participants were sent information sheets and provided written informed consent before participation.

## Results

Of the 33 participants contacted, the interviews were conducted with 25 consenting participants (14 male and 11 female) lasting an average of 50 min per individual. Eight opted out; two patients for reasons of disinterest and six physiotherapists gave scheduling issues as reasons for opting out. However, the response rate was 81% and 70% for patients and physiotherapists, respectively. Details on demographics are shown in Table 1.

**Table 1:** Characteristics of the study population

Characteristics (*n* = 25)	n (%)	Mean (SD)
Sex		
Male	14 (56)	
Female	11 (44)	
Diverse	0 (0)	
Age (in years)		42.8 (SD 11.47)
Physiotherapists (*n* = 12)		
Years of experience with low back pain		
Up to 10 years	9 (75)	
> 10 years to 25 years	3 (25)	
Qualification		
BSc/BMR(PT)	8 (67)	
M.Sc	3 (25)	
Ph.D	1 (8)	
Areas of Interest		
Cardiopulmonary	2 (17)	
Community physiotherapy	3 (25)	
Ergonomics and Occupational	1 (8)	
Geriatrics	1 (8)	
Neurology	1 (8)	
Musculoskeletal	5 (42)	
Paediatrics	3 (25)	
Women's health	1 (8)	
Work setting		
Primary health care	1 (8)	
Teaching hospital and federal medical centres	5 (42)	
Specialist hospital	3 (25)	
Physiotherapy training institute (university)	3 (25)	
Home and community physiotherapy	1 (8)	
Patients (*n* = 13)		
Work status		
Paid work	6 (46)	
Self-employed	5 (39)	
Retired	2 (15)	
Experience with LBP (STarT-Back Classification^a^)		
High risk	7 (53)	
Medium risk	2 (15)	
Low risk	4 (9)	

^a^Based on the STarT-Back Tool. Patients who score 0-3 were allocated to the low-risk subgroup, 4-9 but 3 or fewer of the five sub-scale are medium risk, 4 of 5 on the psychological subscale are high risk. Higher scores indicate an increasing complexity of the condition

The perspectives of participants on TBS were captured in five key themes and 14 sub-categories shown in Table 2. The themes are described as, driving impetus for TBS, influencing perceptions with information, turning to TBS as a final recourse, and exploring the primary alternative and Integrating TBS.

**Table 2:** Themes and sub-themes

Themes	Sub-themes
Driving impetus for TBS	Dissatisfaction with the current system
	Cost
	Inadequate hospital resources
	Expectations of Hard touch
Influencing Perceptions with Information	Patients’ knowledge
	Popularity / Use of TBS
	Word of mouth /Recommendation
	PT Perception of TBS
Turning to TBS as a final recourse	Patients fear and desperation
	Exercising caution
Exploring the primary alternative	Preference over Professional physiotherapy
	Helpful TBS Experiences
Integrating TBS	PTs learning from TBS
	Blending TBS and orthodox practice

*TBS *Traditional Bone Setting, *PT *Physiotherapist

### Driving impetus For TBS

This theme focuses on perspectives of physiotherapists and patients. Patents in Nigeria report significant dissatisfaction with the current state of healthcare services. They report encountering a range of challenges, including poorly maintained equipment and high costs associated with accessing medical care exacerbated by the absence of health insurance coverage.

*PatF7: […] too much old equipment.

*PatM4: The money to undergo those tests was an issue, […]*

* Psudonym for participants: PT, Physiotherapist. Pat, Patient. F, Female. M, Male. 7, Participant number

Moreover, patients experienced limited access to healthcare centers, often finding themselves facing long distances to reach healthcare facilities with the centers being few and widely dispersed across the country. Furthermore, there was the issue of dissatisfaction with the professional system.

*PatM4: […] and I had to travel […] up to 100 kilometers away*

*PTf10: There is the likelihood that patient not be satisfied with our intervention, they will go after the treatment to another therapist or TBS.*

Patients highlighted additional concerns regarding the availability of healthcare personnel. They express frustration over frequent strikes by healthcare workers, leading to disruptions in service delivery, as well as the phenomenon of ‘brain drain’ resulting in a shortage of clinicians relative to the patient population. These factors contribute to a sense of frustration and disillusionment among patients seeking medical treatment.

*PatF9: When it started in I was taken to a hospital, but that time in 2015 the doctors were on strike so I had to go to the TBS.*

In addition to structural and personnel-related issues, patients also express dissatisfaction with the type of care received. Many report unmet expectations regarding the delivery of hands-on massage therapy, with physiotherapists failing to fulfil the expectations of strong, hands-on treatment. This perceived lack of proper hands-on treatment further diminishes patients' confidence in the professional healthcare system and drives them to seek TBS.

*PTm9: When you tell the patient to just come once and go the patient might have the impression you don't have a solution to their problem knowing the normal treatment you should do, at times patients even tell other patients what to expect so they come with an expectation.*

As a result of these challenges, patients increasingly turn to TBS therapy as an alternative form of care. TBS practitioners are perceived as offering more affordable and accessible services, aligning better with patients’ desired intervention. This shift towards TBS reflects patients' desire for healthcare options that better meet their needs and preferences in the face of systemic shortcomings within the professional healthcare system.

*PatF11: TBS is by far cheaper, I paid 30, 000 naira for a card and 10 sessions of treatment in the clinic, but this man [TBS] i paid him just 10,000 naira, one-third of the amount used in the hospital.*

## Influencing perceptions with information

This is a mixed theme as it contains aspects focusing on perspective of physiotherapists and patients. Participants highlight a widespread lack of patient awareness and understanding of the condition. Many patients exhibit limited knowledge of LBP, shown by the struggle to grasp the complex medical terminology often used to describe it within hospital settings. This contributes to feelings of apprehension and uncertainty about the condition, influencing patients' perceptions of available treatment options, including physiotherapy services, which some patients are unaware of altogether.

*PatF11: I had never heard that before in my life, those big words were scary, I had to tell them to write it down for me, so I went to Google it out.*

*PTf7: Some patients don't take time to know about their condition. Many times we find low back pain among illiterate [uneducated] patients.*

Conversely, TBS emerges as a widely recognized and trusted treatment option among patients with LBP. Participants observed that patients frequently turn to TBS due to its popularity and familiarity within the community. Positive word-of-mouth experiences shared among patients further bolster the credibility of TBS, with many individuals attesting to the successful management of various conditions through this traditional form of therapy. As a result, patients are often swayed by these testimonials and are motivated to explore TBS as a potential solution for their LBP.

*PatM4: We have our own native trado-medical doctors (TBS) who manage broken bones traditionally, but I have not seen them handle low back pain.*

However, physiotherapists present a contrasting perspective on TBS, expressing scepticism and concern regarding its efficacy and safety. They perceive TBS as potentially exacerbating patients' conditions and cite numerous instances of poorly treated patients who have reported negative experiences with TBS interventions. These firsthand accounts from physiotherapists underscore their apprehensions about the use of TBS as a viable treatment option for LBP, highlighting a stark contrast in perceptions between healthcare providers and patients regarding traditional versus professional physiotherapy.

*PTf8: When they visit these, they come back (from trado-medical treatments) with complications that I have to deal with so it is a barrier, and over the years it was more, but recently the percentage is coming down now it should be about 30% but back then some years ago it was about 70%.*

## Turning to TBS as a final recourse

Perspectives of patients are the focus of this theme. Several participants expressed the view that TBS should only be considered as a viable treatment option when all other professional options have been exhausted. Moreover, participants demonstrate an awareness of their limited understanding of TBS and the potential risks involved.

*PatM13: No. I have not done any other form of treatment because it did not even come into my thought, and no one has advised me to do that so I didn't even consider it.*

Despite acknowledging the potential risks associated with the limited knowledge and expertise of TBS practitioners, participants perceive similar risks within hospital settings. This perception is grounded in the belief that they have already explored various treatment avenues without success. Many are desperate and this high desperation leads them to seek alternatives.

*PatF11: It was like a movie, I was getting crippled gradually and scared. That period, within October 2019 to 2020 November […], some person told me to even try voodoo, even though I didn't like those things I had to go.*

Despite this acknowledgement, they express a willingness to take calculated risks and exercise caution while undergoing TBS treatment. Participants emphasized the importance of being vigilant and taking necessary precautions to mitigate any potential harm or adverse effects associated with TBS therapy.

*PatM5: (...laughs) he said the guy was bedridden for months, what was supposed to be the cure put him in a horrible position. very serious. Yes, it was a fearful one and the fear of it made me stop.*

### Exploring the primary alternative

This theme focuses on patients perspective. Some participants expressed positive views regarding TBS as an effective management approach for their LBP. Many regard it as their preferred and even first-choice treatment option, highlighting the substantial benefits they have derived from TBS sessions. They emphasize that TBS aligns well with their expectations for addressing LBP and has significantly improved their condition.

*PatF11: I prefer the TBS massage more, because the message you are touching the place that hurts. This traditional man tells me how I will feel when he touches the place and it feels just as he says it. He massages it from my neck and I feel it in my waist, the exact area where I am having the pain starts responding.*

Moreover, participants who initially had reservations about TBS report undergoing a transformative shift in perspective after experiencing positive outcomes. These individuals have become staunch advocates for TBS, citing their own firsthand experiences as compelling evidence of its efficacy. They recount instances where professional physiotherapy treatments, including multiple sessions of physiotherapy and even surgical interventions, failed to provide relief, whereas TBS proved instrumental in alleviating their symptoms.

*PatF11: …I was first afraid. My neighbor initially told me to go traditional, so I asked the clinician in the first hospital if it was possible to fix this and traditional massage, he said no, because since it is a nerve and spinal issue they might touch something that would spoil things. […] But when I visited these hospitals, tried the voodoo and the rest it didn't work then finally I decided to have this treatment. Despite the fear, because I tried other things and they didn't work I became open to other methods of treatment.*

Participants attribute the success of TBS to several key factors, including its proximity to their communities, the personalized care provided by practitioners, and the empathetic approach adopted during treatment sessions. They underscore the importance of the positive experiences they have had with TBS practitioners, which have not only led to subsequent visits but also spurred them to advocate for TBS within their social circles.

*PatM4: Yes, they used to tell us and give us hope, the possibility to get better. they have human sympathy; they advise us sometimes that helps*

### Integrating TBS

This theme focuses on patients. From those interviewed, some participants in the study advocate for the integration of TBS into mainstream medical practice in Nigeria, citing its significant role in facilitating the recovery of many patients. They emphasize the role TBS might play in patient care, particularly in situations where there is a shortage of personnel and limited access to medical equipment. Others opined that the physical touch and massage therapy provided by TBS practitioners could offer valuable support to patients, complementing the services provided by professional physiotherapists. Furthermore, participants stress that while empirical evidence may be lacking, anecdotal evidence should not be discounted as a basis for learning and integration of TBS into mainstream healthcare. This could be done via training of TBS practitioners by professional physiotherapists and training of professional physiotherapists by TBS, open communication between TBS and professional physiotherapists. They highlight potential of TBS in situations where professional physiotherapy may be limited or unavailable.

*PatF11: There should be a collaboration between them, it is not everything science can explain, like my pastor says Satan cannot be seen in a microscope. As some hospitals do not have machines these strong hands can be used instead.*

*PatF11: this girl (physiotherapist) was doing the traditional massage because she is from the area where TBS is learned, she mixed traditional massage and medical care. So that is why I say if you can add traditional massage to their medical it would be excellent.*

Patients interviewed expressed a belief that physiotherapists should be open-minded and not dismiss TBS outright, as they believe TBS had positive contributions to make in patient care. They highlight the potential benefits of incorporating TBS into the healthcare system, drawing parallels with the acceptance of Chinese medical practices as alternative options. Patients argue that TBS could similarly be considered a mainstream alternative in Nigeria.

*PatM1: […] there are two parallel lines, they threw away our traditional practices, there is a lot in it that complements ‘professional’ medicine, and clinicians cannot and should not condemn it.*

## Discussion

This study aimed to explore the perception of Nigerian patients and physiotherapists about practice of TBS. Findings from this study illuminate the complex interplay between patient beliefs, community perceptions, and professional opinions in shaping healthcare-seeking behaviours for LBP management.

Participants' perspectives highlight a nuanced understanding of TBS as an alternative treatment option. While recognizing the potential risks and uncertainties involved, they view TBS as a viable option when professional physiotherapy treatments have failed to meet their expectations. Studies have shown that patients' expectations are key drivers for their choice of treatment and has a significant influence in the outcome [43–45]. These expectations encompass interventions that heavily involve therapist-guided techniques such as manual therapy, thrusts manipulation, and massage, closely aligning with the intervention methods of TBS [46]. A study conducted by Oyebola et al. [22] who surveyed 165 TBS practitioners in western Nigeria, revealed a similar pattern of practice that resonates with practitioners in other regions like Liberia, Mali, East Africa, India, and China. This widespread practice contributes significantly to the growing preference for TBS. One pressing issue is the dwindling resources due to brain drain- referring to the migration of highly skilled scientific professionals from less developed countries to more developed nations, driven by the pursuit of better living conditions, higher salaries, improved quality of life, political stability, and access to superior educational opportunities [47]. Over the past five years, the number of practicing physiotherapists have halved, and fewer new physiotherapists been registered, bringing the ratio of practising physiotherapists to 0.06 per 10,000 creating a substantial manpower shortage [48, 49]. This widely differs from other countries like the US with a ratio of about 7 practicing physiotherapists per 10,000 population, Germany with 24.50 practising physiotherapists per 10,000 population and the UK with a similar healthcare model, with 9.81 practising physiotherapists per 10,000 population [50–52]. These factors, combined with affordability and proximity, contribute to the increasing popularity and patronage of TBS as an alternative treatment option in Nigeria.

Participants' narratives and experiences highlight the importance of individualized care, positive patient experiences, and tangible improvements in symptom management, all of which contribute to the enduring appeal and advocacy of TBS within the realm of healthcare for LBP. This is in line with previous research that highlights the influence of factors like previous treatment experiences, education level, therapeutic freedom and time spent with patients on choice of clinical care [48, 49, 53].

In this study, most patients had a bachelor's degree as their highest educational qualification, which could have reduced chances of bias due to literacy level and influenced the sincerity of their openness to considering TBS treatment. This highlights the significance of patient autonomy and emphasizes the necessity for thorough healthcare decision-making processes.

Distinct perspectives could be seen between physiotherapists and patients, while patients gravitate towards TBS based on its cultural significance and perceived effectiveness, physiotherapists remain cautious, emphasizing the need for evidence-based interventions and comprehensive care approaches to address the multifaceted nature of LBP. They point to extreme cases of ‘debilitation outcomes’ and ‘complications’ experienced by them in the clinics viewing them as unacceptable [24–26]. However, it is evident from patient interactions that only those with poor outcomes tend to return to the physiotherapists. Many patients who have experienced positive results have not revisited the physiotherapists. This aligns with the concept of recency bias, where positive patient experiences reinforce perceived effectiveness, while physiotherapists' encounters with complications foster skepticism. Some attribute this to recognizing the perceived efficacy of TBS and feeling no further need for physiotherapist intervention, while others have sought TBS treatment because they perceived no relief from the physiotherapists. There is also the factor of resolvable acute cases, which would resolve with or without treatment. Such cases might also be attributed to TBS and might contribute to the perception [54].

A more balanced view was also seen in this study, while some participants believe that the physiotherapists need support in terms of manpower, TBS could not work independently leading to participants advocating for a more inclusive approach to healthcare delivery in Nigeria, one that acknowledges and incorporates the contributions of traditional healing practices such as TBS. Studies on integrative medicine and traditional care, have shown that despite controversies and challenges in terms of evidence, there is good chance for fostering interprofessional collaboration with mainstream medical practices. In line with findings from this study, this might mean engaging in in-depth conversations at the level of the practitioners associations of physiotherapy and TBS to define and clarify specifics related to the scope of TBS intervention, communication to reach an equal understanding with practitioners and drawing TBS together for basic education [55–57].

They stress the importance of collaboration between professional healthcare providers and TBS practitioners, working together to ensure that patients receive the most comprehensive and effective care possible. This is seen in a recent study by Onyemachi et al. [19], where the feasibility was seen for integration of TBS by providing formal training. One step towards collaboration is understanding the phenomenon from varying perspectives [53].

This study emphasizes the importance for physiotherapists to enhance open communication with patients and recognize the potential of TBS by either incorporating it into treatment or at least discussing it with patients. To do this, they need some realistic and unbiased information about the profile, intervention and methods of TBS intervention to ensure these practices are within the boundaries of safe and best available evidence [27, 58]. When provided with more information, patients are likely to benefit more from and approach TBS with greater caution [59].

This study adhered to a rigorous systematic method for recruitment and analysis thoroughly described in published literature [35]. One limitation of this study was a secondary analysis, however, relevant findings were highlighted. Due to the method used, care should be taken when generalising results to other countries. However, since TBS is similarly practised in other countries parallels from these findings could be drawn and contextually tailored.

Furthermore, as the study employed a qualitative design where representativeness was not the primary objective, the sample size is deemed adequate. Although, subjective responses from the interviews reveals that all patients had some knowledge and/or experience with TBS, the extent and frequency of participants' exposure to TBS were not documented, which may be seen as a limitation but is characteristic of qualitative research and allows for open interpretation of participants perspectives. Given the qualitative nature of the study, the researcher’s subjectivity, beliefs, and experiences likely influenced the analysis, which is an inherent and valuable aspect of qualitative research [60, 61].

## Conclusion

This study revealed divergent views on the efficacy, safety, and cultural significance of TBS compared to professional physiotherapy interventions. Patients expressed trust in TBS for its accessibility, affordability, and perceived effectiveness in providing relief from LBP symptoms. However, concerns were raised regarding the lack of scientific evidence, standardized practices, and potential complications associated with TBS procedures. In contrast, physiotherapists emphasized the importance of evidence-based practice and patient education in promoting safe and effective LBP management. The study findings underscore the need for open conversation, bilateral learning and fostering integrative care for the benefit of the patient. Clinical insights derived from this study provide valuable guidance for communication and self-awareness for physiotherapists in Nigeria and other regions with a similar healthcare landscape, informing culturally sensitive and evidence-based approaches to LBP management. Given Nigeria’s healthcare challenges, particularly in rural and underserved areas, integrating TBS within the formal healthcare system could expand access to musculoskeletal care while mitigating risks through training and regulation. This calls for open dialogue, interprofessional collaboration, and structured training programs that bridge traditional and modern practices. Future research could explore the feasibility of such integration, ensuring culturally sensitive, safe, and effective LBP management.

## Supplementary Information

Supplementary Material 1. APPENDIX: Themes, Subthemes and Codes. Legend: TBS-Traditional Bone Setting, LBP-Low Back Pain, PT-Physiotherapist.

## Data Availability

All data supporting the findings of this study are available within the paper and its supplementary information.

## Abbreviations

MSK: Musculoskeletal
LBP: Low back pain
PIP: Psychologically Informed Physiotherapy
SC: Stratified Care
PT: Physiotherapist
TBS: Traditional bone setters
