# Supplementary material for: Traditional bone setting in Nigeria from the perspectives of patients and physiotherapists— clinical insights for low back pain management
Source: BMC Complement Med Ther. 2025 Jul 3;25:229. doi: 10.1186/s12906-025-04966-z (PMC12224792; doi:10.1186/s12906-025-04966-z)
Supplement: Supplementary file 1 — Supplementary Material 1. APPENDIX: Themes, Subthemes and Codes. Legend: TBS-Traditional Bone Setting, LBP-Low Back Pain, PT-Physiotherapist. [file 12906_2025_4966_MOESM1_ESM.docx]

APPENDIX 1: Themes, Subthemes and Codes

| **Quotes** | **New Codes** | **Themes** |
| --- | --- | --- |
| *PatM3: I had received treatment for some years until 2016, I was still in lots of pain after physiotherapy and I was contemplating travelling abroad for treatment or alternative care.*  *PatM4: Drugs, physiotherapy, I am not completely relieved, I just got tired of going to the hospital. because I have been going for physiotherapy for the past 2-3 years.*  *PTf10: There is the likelihood that the Patient not be satisfied with our intervention,they will go after the treatment to another therapist or TBS.*  *PatF11: I did visit the TBS because i didn't see any 'head and tail' in that physiotherapy own they were doing, i had some more sessions but i abandoned it, all the ones i did there was no improvement*  *PatM8: Very poor, the infrared light they use on me everyone else has to wait until they are done with me, they have two and one is bad. So, if I needed it for 10 mins it might be cut down to two or three minutes so that others can also use it.*  *PTm9: when you tell the Patient to just come once and go the Patient might have the impression you don't have a solution to their problem knowing the normal treatment you should do, at times Patients even tell other Patients what to expect so they come with an expectation* | Dissatisfaction with current system | Driving impetus for TBS |
| *PTf4: You have to explain to them, once the patients feel dissatisfied they would end up going for a massage and traditional bonesetters.*  *PatM8: I thought by now I should have been fine…I feel the physiotherapy and medication weren't doing anything to help my situation…I just felt I wasted my money and time,*  *PTf4: ‘Then for the Patient, … they might not be satisfied because they are expecting more from you’…if you don't explain why you have to do that and you just do it they won't feel happy. They feel they need some form of hard touch, strong touch.*  *PatF1: I wish it can go, I wish my pain can go… I think it will need some kind of strong force for it to go, maybe I should like a fall or something to put it in position’*  *PatF1: the treatment I have received so far like the taking of drugs all those pain killers I have taken, I would take the pain killers and it would relieve me but after some time the pains will come up again, even for the massage before few weeks or days it relieves but comes up again* | Expectations of Hard touch |  |
| *PatF7: In our health sector, too old people are there, and too much old equipment. there should be an upgrade from the government*  *PatF9: when it started in i was taken to a hospital, but that time in 2015 the doctors were on strike so i had to go to the traditional bonesetters.* | Inadequate hospital resources |  |
| *PatF9: Physiotherapy generally is expensive that is a problem*  *PatF9: i would love find cheaper alternatives like to buy the device, …that massaging machine and i would like to but it to use myself…rather than going to the hospital.*  *PTm9:it is possible they might also accept PT treatment if it costs less but they need to be educated, in such a situation one has to spend more than that 10 minutes to convince them.*  *PatF11: TBS is by far cheaper, i paid 30,000 naira for a card and 10 sessions of treatment in the clinic, but this man i paid him just 10,000 naira, one-third of the amount used in the hospital.*  *PatM4: The money to undergo those tests was an issue, like the MRI i did, before i got the money to do that, it was over 74,000 naira and i had to travel up to 100 kilometers away, i sourced for funds from here and there.* | Cost |  |
| *PTf7: some patients don't take time to know about their condition. many times we find low back pain among illiterate [uneducated] Patients more, if we can make it easier for the common uneducated man it would help*  *PTm5: i can remember that there are quite a lot of patients when i am treating and if they talk about back pain../some do not even know what back pain is they will tell you they have pain around the waist, waist pain, so to even tell them that back pain is pain affecting a certain level to a certain level becomes and issue*  *PTf4: most patients are not aware of Physiotherapy, it's not like their first or second contact, they usually come when it's very late so that means we would just be dealing with the last stage. I don't know how much of a result we would get at that stage of care.*  *PatF11: i had never heard that before in my life, those big words were scary, i had to tell them to write it down for me, so i went to google it out, when i read it online i felt the experience i got was not similar to what i read,*  *PatF9: many people do not know about physiotherapy if i had known earlier since that 10 years ago. i just heard about physiotherapy last year* | Patients’ knowledge | Influencing Perceptions with Information |
| *PatM2: Oh yes, if i have low back pain and i want to go to a place someone who has the same condition and have visited that same place before my discourage me and say ' those ones don't treat you well', that can, of course, affect my expectation. they might advise you positively too and that would boost my confidence to do what they ask*  *PTm5: most people prefer to go to bonesetters and the likes because the people around them would have told them that this is what someone used and the person got better so*  *PatM5: there have been many of them, even at a point i called trado-medical massages that straighten bones to pull my back with their physical strength.*  *PatF11: yes, the state hospital, all of us. they were even complaining. it was one of the patients there that suggested traditional massage to me, we all spoke together and they said since they are not seeing any benefits from this treatment maybe we should try the traditional method, and another lady introduced some supplement drugs but i didn't it because i was pregnant*  *PatF11: yes, but an elderly man there spoke and said i should try [tradomedical] massage. so i just keyed in. though the hospital discourages us from doing it.*  *PatF11: One of the men in the clinics, a patient there said he had experienced it, that a girl comes to his house to massage him very well, he said it was good but she was an apprentice and then she left so things deteriorated. this girl was doing the massage because she is from the area where traditional massage is learned, she mixed traditional massage and medical care. so that is why i say if you can add traditional massage to their medical it would be excellent. Even when he was telling us,he said he called the girl again and begged her to come back, she said she was living in another state now,so we all thought of contributing for her transport fare, 5 of us she would attend to and then we share the cost among ourselves* | Word of mouth /Recommendation |  |
| *PTf8: when they visit these, they come back (from trado-medical treatments) with complications that i have to deal with so it is a barrier, and over the years it was more, but recently the percentage is coming down now it should be about 30% but back then some years ago it was about 70%. it makes their treatment*  *PatM2: yes, when [the physiotherapist] talks about alternative approaches, in most cases he has not got the training that allows him to condemn a different approach because he does not understand what it is about, it is beyond his scope*  *PatM2: So when clinicians avoid trado-medical treatment it is ignorance camouflaged by arrogance. Orthodox practices based on research are functional, but i believe there are good practices they know nothing about because they have no training in that area and cannot say anything about it. The same way a herbal medical practitioner cannot recommend or condemn orthodox practices. They share similar competency and similar vulnerabilities if they know their onus they can do a lot of help and you might believe they are better than the orthodox* | PTs Perception of TBS |  |
| *PatM4: we have our own native trado-medical doctors who manage broken bones traditionally, but i have not seen them handle low back pain.*  *PatF1: I have taken medications, pain killers and I have gone for traditional /native massage*  *PatF9:Yes, I went for trado-medical massage for 3 weeks*  *PatM5: there have been many of them, even at a point i called trado-medical massages that straighten bones to pull my back with their physical strength.* | Popularity / Use of TBS |  |
| *PatF7: because i feel my case is not as terrible as that, only the very terrible cases go to the TBS. I felt it was something that could be handled by the hospital*  *PatM8:no not at all, people have been trying to talk me into it but i am afraid because i know it has to do with the spine i do not want to complicate things and take the risk. I think it is risky, i have not been a fan of trado-medical cure*  *PatM10: well i am prepared for anything, even if it will be TBS or surgery*  *PatF11: It was like a movie, i was getting crippled gradually and scared. that period, within October 2019 to 2020 November between you and me, i now know the reason why people commit suicide. It was horrible. Some person told me to even try voodoo, even though i didn't like those things i had to go.* | Patients fear and desperation | Turning to TBS as a final recourse |
| *PatM5: (...laughs) he said the guy was bedridden for months, what was supposed to be the cure put him in a horrible position. very serious. Yes it was a fearful one and the fear of it made me stop*    *PatF6: No traditional massage because we don't know much about that and it may be harmful. No local massage. we know about it but we don't believe in it.*  *PatM4: i didn't try traditional treatment, i don't know much about it*  *PatM13: No i have not done any other form of treatment because it did not even come into my thought, and no one has advised me to do that so i didn't even consider it* | Exercising caution |  |
| *PatF11: i prefer the TBS massage more, because the message you are touching the place that hurts. this traditional man tells me how i will feel when he touches the place and it feels just as he says it. He massages it from my neck and i feel it in my waist, the exact area where i am having the pain start responding.*  *PatM5: i can't remember clearly, but i was visiting the hospital often. some times when it comes i would want to manage it, because i hate going to the hospital*  *PatM8: i plan to get another treatment place, somewhere they will have my time and i can explain to because in the hospital i was attending it was more like because i have paid my money they are just attending to me because that is what i paid for, they didn't take any particular interest.*  *PatF11: The man's hand was wonderful, he did some massage and there was this thing he did, he asked two of his sons to help, one held my two legs and the other held two of my hands, and they were pulling in opposite direction. while this was happening the man put his leg on my back and was pushing downwards gradually avoiding the pregnancy, he pushed towards the right side where the pain was more, at first i felt pains but after that i felt i could stand erect. he did it three times and i was very okay.* | Preference over Professional physiotherapy | Exploring the primary alternative |
| *PatM2: ‘there is a lot in it (trado-medical care) that complements orthodox medicine’…I can manage my pain better.*  *PatF11: Yes, i was first afraid. My neighbour initially told me to go traditional, so i asked the clinician in the first hospital if it was possible to ix this and traditional massage, he said no, because since it is s nerve and spinal issue they might touch something that would spoil things. so when my mum and others suggested the same thing i said no i wasn't going. But when i visited these hospitals, tried the voodoo and the rest it didn't work then finally i decided to have this treatment. Despite the fear, because i tried other things and they didn't work i became open to other methods of treatment.*  *PTm5: most people prefer to go to bonesetters and the likes because the people around them would have told them that this is what someone used and the person got better.*  *PatF1: because after the physiotherapy i also went for the traditional massage, that was where i got better. The traditional massage helped more.*  *PatF11: no i didn't have any message from her, but when she called me this year and told me she has relocated, she asked about me and the baby, i told her i lost the baby, after all she said How am i feeling? that was when i told her i went for a massage and i saw great improvement, then there was a pause, she now asked if i was feeling better i said yes, and the pains are no longer there. Formerly i cannot walk from the bedroom to the toilet without crying out in pain, now i can walk a long distance without pain*  *PatM4: yes, they used to tell us and give us hope, the posibility to get better. they have human sympathy, they advise us sometimes that helps*  *PatM10: all my muscles were stiff when i first visited, they used the light on me and it relieved me in addition to the exercise. i was doing the exercise too. but he said i might have to do surgery on it. maybe i was using too much force on the back. because i was desperate to walk properly with it. they were even surprised that how am i moving with it i said it was the work of God* | Helpful TBS Experiences |  |
| *PatM1: exactly, for example, massage in the orthodox massage there is also traditional massage, they are experts that use herbs to solve these problems. There are two parallel lines, they threw away our traditional practices, there is a lot in it that complements orthodox medicine, and clinicians can not and should not condemn it.*  *PatM2: yes, that is why when i think of traditional practices they are not to be thrown aside, there is the mentality that you should still on working in the farm or so even if you have pain. So traditionalists are not quacks because they have received the training.*  *PatM2: yes, that's what the Chinese do, theirs are highly developed and its practised side by side with orthodox doctors, these ones go back to study Chinese medicine and find out there are a lot of aspects that are highly effective, they also advise their Patients on these things, it is unfortunate that the African traditional medicine was thrown out, there are a lot of things that could complement orthodox medicine in there.*  *PatF11: this girl (physiotherapist) was doing the traditional massage because she is from the area where TBS is learned, she mixed traditional massage and medical care. So that is why I say if you can add traditional massage to their medical it would be excellent.* | PTs learning from TBS | Integrating TBS |
| *PatF11: There should be a collaboration between them, it is not everything science can explain, like my pastor says satan cannot be seen in a microscope. as some hospitals do not have machines these strong hands can be used instead. with his hand he opens a closed cervix before me. So some things the hospital cannot handle, these know where and where to touch.*  *PatF11: you spoke a lot about your experiences with a private clinic, government hospital, traditional treatment and you saw improvement finally with traditional care and you suggest they should combine traditional care and medical care for maximum benefits.*  *PatM2: Unfortunately, these practices were maligned and condemned, Assuming a structure was build around it like the way they did orthodox as the Chinese did theirs we should have both on the table and co-operate to help patients.* | Blending TBS and orthodox practice |  |
